# Incisional metastatic breast carcinoma deposit in a total knee replacement presenting as peri-prosthetic joint infection—a case report

**DOI:** 10.1093/jscr/rjac139

**Published:** 2022-03-31

**Authors:** Aysha Rajeev, Ahmed Elzawahry, Simren Rakhra, Kiran Singisetti

**Affiliations:** Queen Elizabeth Hospital, Gateshead Health Foundation NHS Trust, Sheriff Hill, Gateshead NE9 6SX, UK; Queen Elizabeth Hospital, Gateshead Health Foundation NHS Trust, Sheriff Hill, Gateshead NE9 6SX, UK; Queen Elizabeth Hospital, Gateshead Health Foundation NHS Trust, Sheriff Hill, Gateshead NE9 6SX, UK; Queen Elizabeth Hospital, Gateshead Health Foundation NHS Trust, Sheriff Hill, Gateshead NE9 6SX, UK

## Abstract

Cutaneous metastasis from the primary breast carcinoma occurs when the disease is wide spread and can present as skin infection especially in a previous well-healed scar. If the secondary deposit is over a total knee incisional site it can mimic peri-prosthetic joint infection. We report a rare and unusual case of a woman who presented with clinical signs and symptoms of a peri-prosthetic total knee replacement which on biopsy turned out to be cutaneous metastasis from a previously treated breast cancer. Chronic granulation tissue in a total joint incisional scar may present as peri-prosthetic joint infection. A good history taking and clinical examination with specimens from the skin lesions send for both microbiology and histopathology is recommended to arrive at an early and accurate diagnosis.

## INTRODUCTION

Skin metastasis of a primary tumour is uncommon and the incidence is about 5% [1]. The most common sources of cutaneous metastases are breast, colorectal and melanoma [2]. It may present as a solitary or multiple nodules and sometimes tend to ulcerate [3]. They can mimic inflammatory dermatitis or cellulitis [4]. We report a rare and unusual case of metastatic infiltration of total knee replacement scar presenting clinically as peri-prosthetic joint infection.

## CASE REPORT

**Figure 1:**
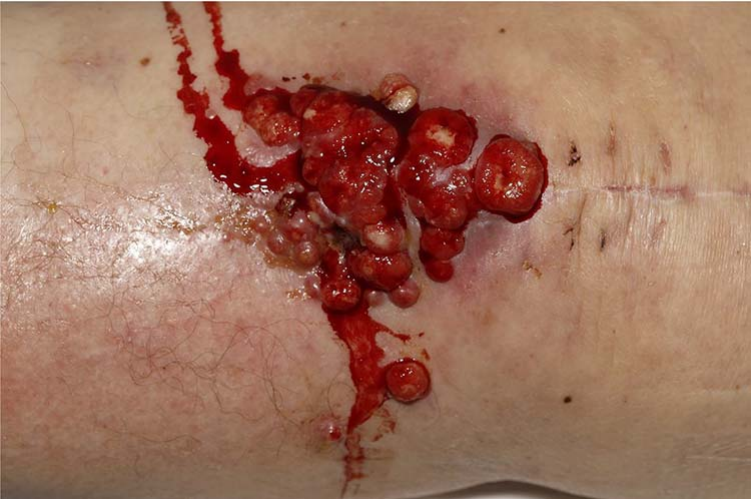
Multiple granulating areas with discharge along the scar of a previous total knee replacement.

**Figure 2:**
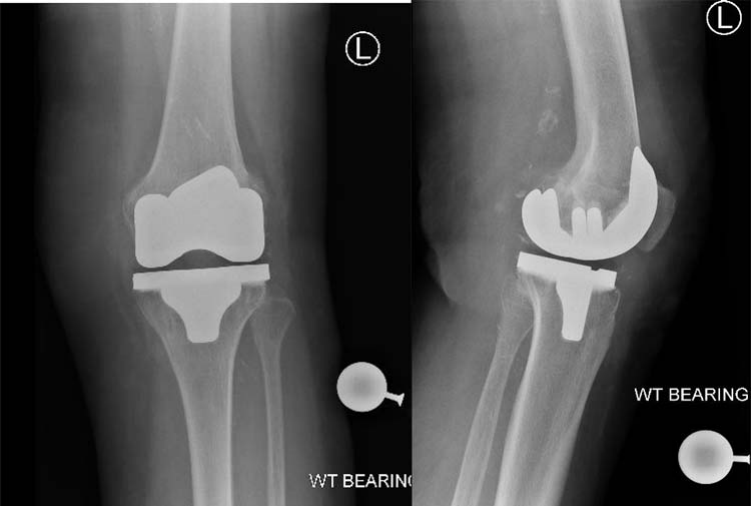
Post-operative radiographs showing satisfactory position of prosthesis for left total Knee replacement.

A 83-year-old lady, previous history of breast cancer treated with mastectomy and chemotherapy and in remission was referred to the orthopaedic outpatient clinic by her general practitioner (GP) with a 3-week history of a superficial skin lesion with multiple granulating areas with discharge along the midline scar of a previous total knee replacement, Fig. 1.The primary total knee replacement was done 24 months ago in February 2018, wound healed well with no evidence of infection. The range of motion after the knee replacement was from 0° to 110° of flexion, Fig. 2. She also had recent urinary tract infection (UTI) with pseudomonas and has been on long-term oral antibiotics for recurrent UTI in the past for the last 12 months. Wound swabs from the total knee replacement scar done by GP showed pseudomonas growth.

On examination there were multiple sprouting granulation tissues along the midline scar of previous TKR with mild discharge. Movements of the left knee were restricted with 10° of fixed flexion deformity and further only 30° of flexion possible. Her inflammatory markers showed a WCC 14.9 and CRP 120.9.A initial diagnosis of peri-prosthetic joint infection was made and was taken to theatre. Under general anaesthesia the knee was aspirated which revealed sero-sanguineous fluid. The wound is then explored and multiple tissue samples were send for both microbiology and histopathology. The extensor mechanism was found to be intact. Intra-operatively we felt that there was no obvious deep joint infection or communication, hence deep joint was not explored. The revision of TKR was not considered as there was no evidence of deep infection.

**Figure 3:**
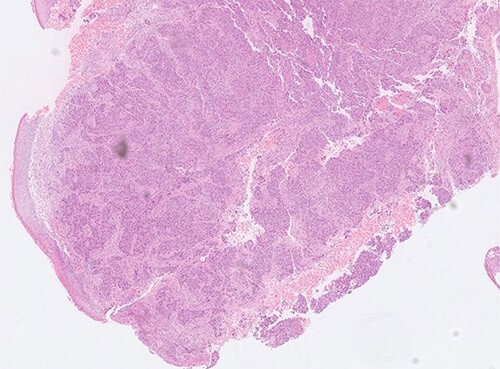
Histopathology slide showing poorly differentiated adenocarcinoma with sheets and nests of malignant cells.

**Figure 4:**
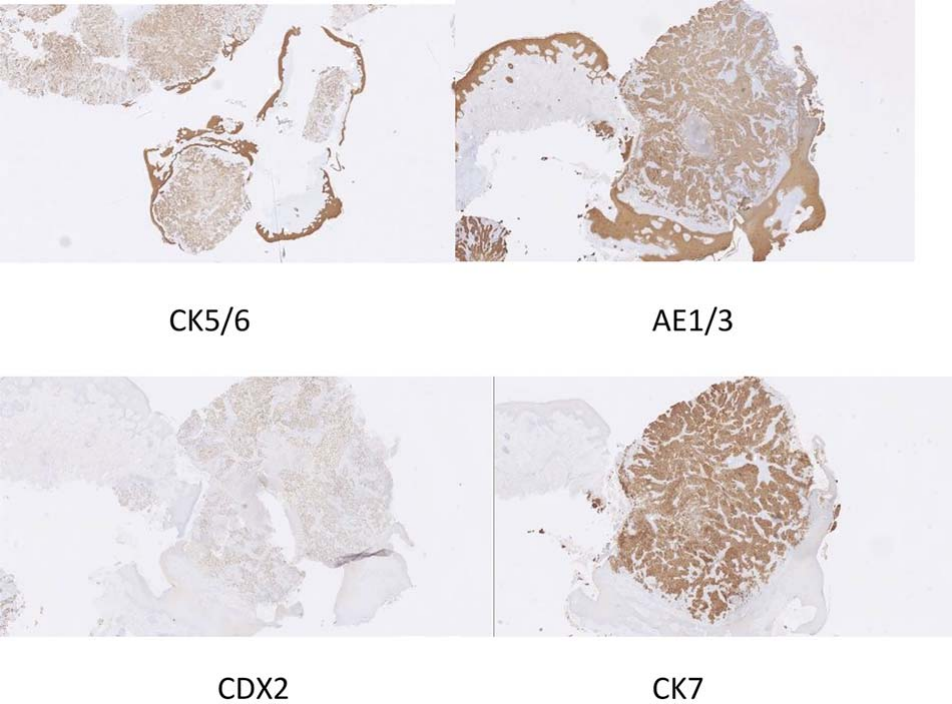
Immunohistochemistry slide showing poorly differentiated adenocarcinoma compatible with breast carcinoma.

Superficial skin debridement and wound closure was done. The knee aspirate did not grow any organisms after extended cultures. The tissue samples have grown pseudomonas sensitive to piperacillin and gentamicin. She was commenced on 100 mg piperacillin intravenously every 8 hours and one dose of 160 mg of gentamicin after consulting with the microbiologist and checking the renal function. The histopathology showed poorly differentiated adenocarcinoma with sheets and nests of malignant cells and central tumour necrosis, Fig. 3. Immuno-histochemistry showed that tumour was strongly positive for CK5/6, AE1/AE3, CDX2 and CK7 which is suggestive of a metastatic poorly differentiated breast carcinoma, Fig. 4.

The patient was referred to oncology for chemotherapy and radiotherapy. She was also referred to plastic surgery as advised by the oncology team for local control of the disease in addition to adjuvant chemotherapy and radiotherapy. She underwent wide local excision and skin flap cover. But unfortunately she passed away after two months of commencement of chemotherapy.

## DISCUSSION

Cutaneous metastatic malignancy is defined as a neoplastic lesion affecting the dermis or the subcutaneous tissue that originates from another primary tumour [5]. Cutaneous metastasis from breast cancer often occurs when the disease is wide spread. It may be resistant to conventional radiotherapy and chemotherapy because of extensive angiogenesis [6]. The mechanisms by which metastasis to the skin occurs as a result of lymphatic or haematogenous spread of tumour [7]. The spread from breast carcinoma to extremities may suggest intra-arterial embolic spread, whereas metastases to the chest wall is suggestive of dissemination by lymphatic vessels or by veins [8]. In our case the metastasis from the breast cancer spread to the lower limb extremity and presented as discharging sprouting granulation tissue.

The clinical presentation may vary and present as multiple discrete nodules or ulcerations [4]. Metastatic skin lesions from breast cancer are usually asymptomatic and appear as firm, pink to red–brown nodules on the chest close to the primary tumour. The common presentations include multiple telangiectatic papules, peau d’orange and carcinoma en cuirass [7]. In our case the patient was in remission from previous breast cancer treatment and the clinical presentation was atypical as discharging sinus with infected red granulation tissue suggestive of peri-prosthetic joint infection.

The primary tumour spreading to the skin depends on age and gender. In adult women the primary tumour is usually breast cancer, melanoma, colorectal cancer and lung cancer. In men, the most common primary is lung cancer, melanoma, colorectal cancer and prostate cancer. In children neuroblastoma and rhabdomyosarcoma are the commonest ones [7]. Our patient was an adult female with breast cancer as the primary source.

The differential diagnosis of cutaneous metastases is cysts, inflammatory lesions, herpes zoster, lupus erythematosus or condylomata [7]). Biopsy of a suspected skin metastasis should confirm or exclude malignancy. The majority of skin metastatic tumours are adenocarcinomas [9]. Immuno-histochemical studies are needed to diagnose a poorly differentiated carcinoma. In our case the tumour was poorly differentiated carcinoma and the diagnosis is confirmed by immuno-histochemistry.

The average time interval between the diagnosis of cutaneous metastasis and death is about 9.4 months [4]. Even though the presentation of breast cancer skin metastasis is late the prognosis is better than those patients with metastasis to lung, liver, bone and brain [10]. Our patient died within 2 months of clinical presentation.

## CONCLUSION

A high index of clinical suspicion and meticulous attention to past medical history including previous cancers especially patients in remission and those treated for malignancy in the past are needed to diagnose cutaneous metastasis. In rare cases, skin metastasis of primary tumours such as breast, colon, lung and prostate can manifest atypically as septic or peri-prosthetic joint infection.

## CONFLICT OF INTEREST STATEMENT

None declared.
